# Animal Toxins: How is Complexity Represented in Databases?

**DOI:** 10.3390/toxins2020262

**Published:** 2010-02-21

**Authors:** Florence Jungo, Anne Estreicher, Amos Bairoch, Lydie Bougueleret, Ioannis Xenarios

**Affiliations:** 1 Swiss Institute of Bioinformatics, Centre Medical Universitaire, 1 rue Michel-Servet, 1211 Genève 4, Switzerland; Email: Anne.Estreicher@isb-sib.ch (A.E.); Lydie.Bougueleret@isb-sib.ch (L.B.); Ioannis.Xenarios@isb-sib.ch (I.X.); 2 Department of Structural Biology and Bioinformatics, Faculty of Medicine, University of Geneva, Switzerland; Email: Amos.Bairoch@isb-sib.ch (A.B.); 3 Swiss Institute of Bioinformatics, Vital-IT Group, 1015 Lausanne, Switzerland

**Keywords:** animal toxin, ArachnoServer, ATDB, ConoServer, database, Tox-Prot, UniProtKB/Swiss-Prot, venom protein

## Abstract

Peptide toxins synthesized by venomous animals have been extensively studied in the last decades. To be useful to the scientific community, this knowledge has been stored, annotated and made easy to retrieve by several databases. The aim of this article is to present what type of information users can access from each database. ArachnoServer and ConoServer focus on spider toxins and cone snail toxins, respectively. UniProtKB, a generalist protein knowledgebase, has an animal toxin-dedicated annotation program that includes toxins from all venomous animals. Finally, the ATDB metadatabase compiles data and annotations from other databases and provides toxin ontology.

## 1. Introduction

Animals have developed different strategies to capture the prey on which they feed and also various means to defend themselves from predators. Venomous animals, such as snakes, scorpions, spiders, jellyfish, hymenopterans (ants, wasps and bees), cone snails, sea anemones, lizards, centipedes, and the platypus are equipped with injection devices (spines, fangs, stingers, hypostomes, spurs, harpoons) that permit the active use of venom in predation. Poisonous animals, such as some mammals, toads, ticks, or worms, which only produce toxic substances for defensive purposes, lack such organs [1]. However, both venomous and poisonous animals have developed a wide-ranging treasure trove of substances designed to imperil potentially threatening organisms in their environment.

Animal toxins are mainly proteins and peptides. Their modes of action are extremely diverse. Many act on the nervous system, affecting ion channels, perturbing glutamatergic transmissions, stimulating transmitter release, *etc.* Others disrupt host cell membrane, behaving as antibacterial, cytolytic, or hemolytic peptides. In addition, a wide variety of enzymes, such as phospholipase A2, proteases, oxidases, sphingomyelinases, *etc.*, are found in animal venom in different taxonomic groups.

In the past 30 years numerous studies on venom proteins have been carried out, leading to the growing evidence that these substances are endowed with remarkable biological properties. Toxins have been used not only in fundamental research as biological tools, particularly to characterize ion channels, but they have also allowed the development of pharmaceutical agents and insecticides [2]. These numerous applications have turned the spotlight on toxins and stimulated new research, leading to an explosion in the number of characterized venom proteins.

To provide the scientific community with animal protein toxin sequences and other relevant information, databases are an absolute requirement. UniProtKB is a well established, general interest knowledgebase centered on protein sequences. In 2002, the UniProtKB/Swiss-Prot team initiated a new annotation program focused on animal toxins, called Tox-Prot. This program aims to systematically annotate venom proteins and proteins produced by poisonous animals that act as toxins [3]. The first step consisted of the annotation of small (<10 kDA) toxin proteins. This initial set included a majority of toxins from spiders, hymenopterans, cone snails and scorpions, as well as a significant proportion of sea anemone and snake toxins.

The Swiss-Prot initiative was followed by the establishment of a few databases centered on toxins. Currently, three additional databases are available on the web. These are ConoServer, ArachnoServer and the Animal Toxin DataBase (ATDB). ConoServer [4] specializes in toxins from marine cone snails (Conus spp.). ArachnoServer [5] focuses on spider venom proteins. ATDB is a metadatabase that collects information from several databases, mostly from UniProtKB, GenBank and other NCBI databases, as well as from databases on snake, scorpion and mollusk toxins that are no longer maintained. The absence of actively maintained databases specific to snake or scorpion toxins is quite surprising given the importance of such toxins as research tools for probing mammalian ion channels.

In this review, we describe how the knowledge on animal toxins is presented in the currently available databases. While it has been proposed that all proteins found in venom be called ‘toxin’, we will consider only those exhibiting toxic activities.

## 2. Sequences

**Figure 1:**
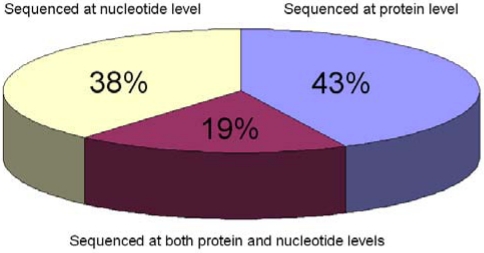
Venom protein entries in UniProtKB/Swiss-Prot (UniProt release 15.10 of 3 November 2009). Out of a total of 3’579 venom proteins, the sequence of 1’375 (38%) was obtained by translation of a nucleotide sequence (cDNA or genomic DNA). 1’528 sequences (43%) were obtained by direct protein sequencing, and 669 toxins (19%) were sequenced at both protein and nucleotide levels.

As is the case for most protein sequences currently available, animal toxin sequences are often obtained by the translation of coding sequences (CDSs) submitted to EMBL/GenBank/DDBJ databases. These translated CDSs are automatically retrieved to populate protein sequence databases, such as GenPept or UniProtKB/TrEMBL. However, a distinctive feature of most polypeptides in invertebrate venoms, as well as a significant part of the non-enzymatic protein components of snake venoms, is that they are small, usually between 1-10 kDa, making them good candidates for classical protein sequencing methods, such as Edman degradation or for MS/MS analysis. As a result, the sequence of 43% of the metazoan toxins in UniProtKB (release 15.10 of 3 November 2009) was obtained exclusively by direct protein sequencing, rather than by translation of a nucleotide sequence (Figure 1).

### 2.1. ArachnoServer

ArachnoServer (http://www.arachnoserver.org/) is a manually curated database that is centered on mature active peptides. Its strategy is to display one toxin sequence per entry. Precursor sequences can be found in the ‘Protein Information’ and ‘Sequences’ sections, and in cases where different precursors give rise to the same mature toxin they are all displayed in the same entry. Conversely, if a single gene encodes two or more active peptides, these are described in two or more separate entries. For example, spider M-zodatoxin-Lt6a and M-zodatoxin-Lt6b are synthesized from a 116 amino acid long precursor, which is subsequently cleaved to give rise to two mature toxic peptides: 33 amino acid long M-zodatoxin-Lt6a and 35 amino acid long M-zodatoxin-Lt6b. Two separate entries exist in ArachnoServer for these active peptides.

‘Sequences’ gives access to the precursor protein sequence, and to the corresponding nucleic acid sequence(s), all in FASTA format. Similarity searches can be launched directly from this topic through various BLAST tools (blastp, blastn, blastx, and tblastn), but alignment tools are not available.

ArachnoServer provides cross-references to several databases, including the EMBL nucleotide data bank, which allows retrieval of the original nucleotide sequence submission. Cross-references to UniProtKB are also found in this section.

### 2.2. ConoServer

Like ArachnoServer, ConoServer (http://www.conoserver.org/) is a manually curated database. Protein sequences, nucleotide sequences and 3D-structures are stored in separate entries. To have a complete view of the current knowledge for a given toxin, users should thus consult all entries. Browsing is facilitated by internal links connecting all entries dealing with the same compound.

Contrary to ArachnoServer, each ConoServer protein entry gives access to a unique sequence. Hence similar, although not 100% identical sequences are shown in different entries, regardless of the origin of the discrepancies. This strategy also applies to nucleotide sequence entries. As a result, a search in the database with the sequence of a mature peptide might retrieve several entries. For instance, such a search with the sequence of the mature conopeptide ABVIA gives five potential precursors within the same species (*Conus abbreviatus*). The origin of these precursors remains an open issue. Differences between protein sequences can be due to a biological process (e.g., precursor maturation leading to shorter peptides, gene duplication, polymorphisms, *etc.*), or a technical problem (e.g., sequencing errors). The source of the data for mature peptides (nucleic acid or protein sequence) is indicated in the entries in ‘Sequence/Sequence evidence’.

A specificity of ConoServer is that it integrates not only natural peptides, but also synthetic ones, including those produced by *in vitro* mutagenesis, and patented sequences. This information is displayed in ‘General information’.

Several useful software tools are provided by ConoServer for sequence analysis, such as ClustalW for sequence alignment and ‘Digest Peptide’ which performs *in silico* digestion with four different enzymes (trypsin, chymotrypsin endoGluC and thermolysin).

Cross-references to GenBank, GenPept or to UniProtKB are provided.

### 2.3. UniProtKB

UniProtKB (http://www.uniprot.org/) is a generalist protein sequence knowledgebase, composed of two sections: UniProtKB/Swiss-Prot, which is manually annotated and reviewed, and UniProtKB/TrEMBL, which is automatically annotated and not reviewed. One of the missions of UniProtKB is to provide users with non-redundant comprehensive data. To meet this requirement, in the UniProtKB/TrEMBL section, all sequences from the same organism that are 100% identical over their entire length are merged into a single entry. In the Swiss-Prot section, this concept is extended to include various isoforms, polymorphisms, fragments, *etc.*, and differences between merged sequences are carefully documented in the entry. Thus sequences encoded by the same gene will be found in a single Swiss-Prot entry, but in separate TrEMBL entries if they differ even by a single amino acid residue.

As a result, several mature toxins can be found in a single UniProtKB/Swiss-Prot entry if they are encoded by the same gene or are derived from the same precursor protein. Looking back at the example of the spider toxin M-zodatoxin-Lt6a/b (see section on ArachnoServer), the corresponding UniProtKB/Swiss-Prot entry Q1ELU8 describes the precursor protein that gives rise to two mature toxic peptides. The cleavage sites used during maturation are annotated in ‘Sequence annotation’ and the sequences of both mature peptides can be retrieved from this section. 

UniProtKB, being protein-centric, does not display the underlying nucleotide sequences in the entries, but cross-references to the EMBL/GenBank/DDBJ databases are provided. 

Close to half of the toxin sequences displayed in UniProtKB come from the translations of CDSs submitted to the EMBL/GenBank/DDBJ nucleotide sequence resources. These CDSs are either generated by gene prediction programs or are experimentally identified. It is thus important to discriminate between predicted and experimentally identified proteins. In UniProtKB a status of protein existence is indicated for each protein. Five such statuses have been defined: 1) evidence at the protein level (e.g., Edman degradation, or MS identification), 2) evidence at the transcript level (e.g., cDNA sequencing), 3) inferred by homology (strong sequence similarity to known proteins in related species), 4) predicted and 5) uncertain (e.g., dubious sequences that could be the erroneous translation of a pseudogene). In November 2009 (release 15.10), close to 99% of all venom proteins from UniProtKB/Swiss-Prot, were identified at the protein (65%) or transcript (34.5%) level.

Several tools are at hand within each UniProtKB entry in the ‘Sequences’ section. They include ‘ProtParam’ [6], ‘Compute pI/MW’ [6], ‘ProtScale’ [6], ‘PeptideMass’ [6,7] and ‘PeptideCutter’ (see ‘Useful URLs’), allowing not only the detailed analysis of the sequence (or subfragments thereof), but also its virtual digestion with close to 40 different enzymes. Last, but not least, protein similarity searches can be performed directly from the entry using BLAST.

In all three databases, search tools are provided that allow to find and retrieve the toxins of interest. It is thus possible to create a customized dataset that can subsequently be downloaded in FASTA format and used for identification by mass spectrometry (see Table 1).

## 3. Post-Translational Modifications

*In vivo* toxins follow the secretory pathway. Thus the first post-translational modification (PTM) they undergo is cleavage of the signal peptide within the endoplasmic reticulum. Many other PTMs occur subsequently and some will have a dramatic effect on toxin function. The formation of disulfide bonds stabilizes the 3D-structure, which often is extremely tight. The addition of simple and complex chemical groups, such as amidation, bromination, hydroxylation, carboxylation, sulfation, *N*- or *O*-glycosylation, palmitoylation, *N*-terminal pyrrolidone carboxylic acid formation, has also been described. Last, but not least, D-amino acid isomerization is thought to be a widely used strategy across the animal kingdom to activate toxins. This has been observed for instance in iota-conotoxin RXIA and spider omega-agatoxin-Aa4B, where the isomerization of a single amino-acid has a potent excitatory effect [8,9].

The description of PTMs in databases is thus essential for a complete picture of toxin biology.

### 3.1. ArachnoServer

**Figure 2:**
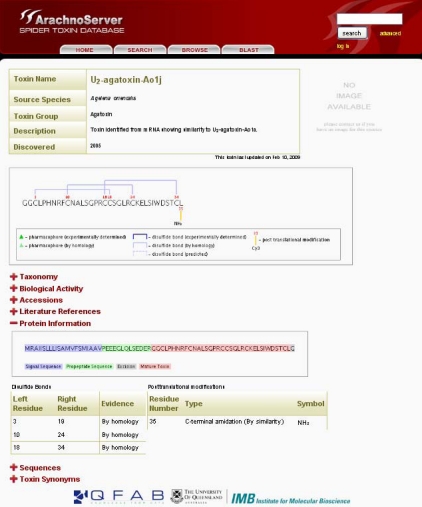
ArachnoServer entry of U2-agatoxin-Ao1j. Mature toxin with three disulfide bonds and amidated C-terminus are graphically shown in the first box. The sequence of the precursor protein is shown in the ‘Protein Information’, signal and propeptide sequences are colored in blue and red, respectively.

In ArachnoServer, information on PTMs, including the position of the modified residue and the level of evidence (experimental or propagated from a homologous protein), is provided in ‘Protein Information’ (Figure 2). The positions correspond to the mature peptide. 

A nice graphical view illustrates PTMs directly on the mature peptide sequence. The modified residue is numbered and a yellow line leads to the PTM in question. Information concerning the experimental evidence is provided for disulfide bridges, which are represented by blue lines whose intensity is variable according to the level of evidence, from dark blue meaning ‘experimental’ to light dotted blue meaning ‘predicted’.

PTMs (C-terminal amidation, D-serine, O-palmitoyl-threonine and pyroglutamic acid) are included in the ArachnoServer search tool and browser, allowing easy retrieval of the modified toxins. Toxins can also be searched on the basis of the number of PTMs they undergo.

### 3.2. ConoServer

**Figure 3:**
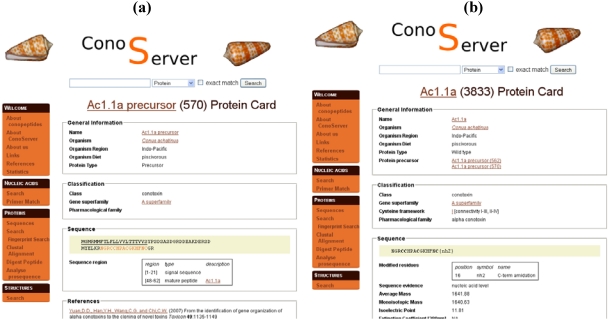
Parts of ConoServer toxin entries. (a) Ac1.1a precursor entry. Signal peptides and mature peptide ranges are indicated in ‘Sequence region’. (b) Mature toxin Ac1.1a entry. The C-terminal modified residue is reported in ‘Sequence’. Disulfide connectivity is indicated in ‘Classification’. A description of the cystine framework and pattern is provided in the documentation of the database available from the homepage (See ‘Useful URLs’).

In ConoServer, the description of PTMs depends upon the displayed sequence, *i.e.*, whether it corresponds to the precursor (Figure 3a) or to the mature toxin (Figure 3b). In precursor entries, the cleavages of signal and propeptides are indicated, whereas other PTMs such as amidation, hydroxylation, or carboxylation of glutamate, essential for the activity of the toxin, are described in the entries corresponding to mature toxins (Figure 3b). The positions of the modified residues, along with the nature of the PTM, are provided, except for cysteines involved in disulfide bonds. These are described with an indication of connectivity, such as I-III, II-IV. There is no information about the evidence for PTMs. The ‘Analyse prosequence’ tool allows the prediction of PTMs, such as signal sequence or propeptide processing, pyroglutamic acid, gamma-carboxylic acid, and C-terminal amidation. Contrary to ArachnoServer, only disulfide connectivity can be searched in ConoServer to the exclusion of all other PTMs.

### 3.3. UniProtKB

**Figure 4:**
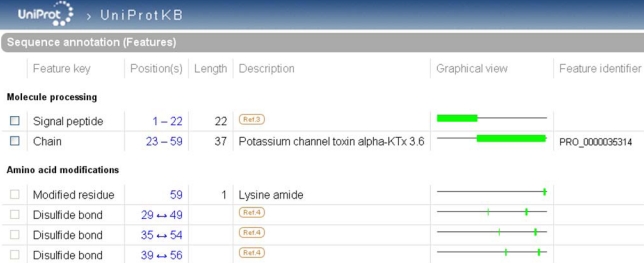
Highlight of the ‘Sequence annotation’ section of the scorpion potassium channel toxin alpha-KTx 3.6 entry in UniProtKB (UniProtKB accession number Q9NII7).

In UniProtKB, PTMs are described in the ‘Sequence annotation (Features)’. The positions of the modified amino acids correspond to the canonical sequence displayed by default in the entry. This sequence can be either the mature peptide or the precursor depending upon data availability. Usually the longest sequence is chosen in order to allow maximal feature annotation. The description of PTMs in this section includes cleavages, such as those of the signal sequence and of propeptides, cross-links, such as disulfide bonds and chemical additions of simple or complex groups. Features can be tagged with one of the three available non-experimental qualifiers: ‘Potential’, ‘Probable’ and ‘By similarity’. ‘Potential’ means that the site has been determined using a prediction program. It may be used, for instance, for a predicted signal peptide cleavage site. ‘Probable’ is added when converging non-experimental evidence suggests the existence of the feature. The data tagged with this qualifier are more reliable than those with ‘Potential’. ‘By similarity’ indicates that experimental evidence was obtained for a homologous protein. The absence of a qualifier characterizes experimentally observed features. In this case, a link is generally provided to the bibliographic source of information (Figure 4). The boundaries of each feature are clickable, which permits not only visualization of the feature in its sequence context, but also retrieval of the subsequence involved (the mature peptide, for instance) in FASTA format ready to use in a BLAST search, for example.

PTMs are included in the UniProtKB search tool also through the usage of specific keywords. Close to 50 keywords have been created for PTMs, which can be found in the document ‘keywlist.txt’ (see ‘Useful URLs’). Among the numerous toxins that are disulfide linked, a particular class is characterized by a structure of ‘disulfide through disulfide knot'. For these small proteins, the keyword ‘knottin’ has been created. Currently 185 of the animal toxins annotated in UniProtKB/Swiss-Prot possess this keyword (release 15.10 of 3 November 2009). 

## 4. 3D-Structure

Due to their pharmacological importance, toxin 3D-structures are extensively studied. Currently, about 15% of the toxins annotated in UniProtKB/Swiss-Prot have an experimentally determined3D-structure, while the overall proportion in the knowledgebase is inferior to 3% (release 15.10 of 3 November 2009). 

3D-structure data are not stored in any of the databases we are discussing in this review, but all of them provide cross-references to 3D-specialized databases. For all toxins whose 3D-structure has been experimentally determined ArachnoServer, ConoServer and UniProtKB provide links to the protein data bank (PDB) which is a repository for experimentally-determined structures of proteins, nucleic acids, and complex assemblies. In ArachnoServer and UniProtKB, toxins that have not been studied at 3D-structure level, but are quite similar to proteins of known structure contain a cross-reference to HSSP (Homology-derived Secondary Structure of Proteins database) [10]. Finally ConoServer provides links to Biological Magnetic Resonance Data Bank (BMRB), a repository for NMR Spectroscopy data on proteins, nucleic acids, and other biomolecules [11].

ArachnoServer and ConoServer also offer various tools to visualize the structure directly from their entries. In UniProtKB, access to this type of tool is provided through a cross-reference to PDBsum [12]. PDBsum summarizes information about each experimentally determined structure in PDB, and provides tools to visualize 3D-structures or to load structures, such as AstexViewer [13], for example.

As already mentioned above (see ‘Sequences’), in ConoServer, all information and tools related to structure are stored in entries separated from those dealing with protein or nucleotide sequences; internal links connect all entries (protein, nucleotide and 3D) dealing with the same compound. 

3D-structure entries can be readily retrieved using the ConoServer search tool which integrates this option. 

In UniProtKB, the keyword ‘3D-structure’ allows the retrieval of toxins with a known structure. 

## 5. Functions and Targets

One of the most striking features of toxins is their high specificity towards a wide range of ion channels, receptors and transporters. This selectivity makes toxins excellent pharmacological probes, as well as lead molecules in drug design [14]. Furthermore, the study of the mode of action of toxins may unravel the function of their targets. This is illustrated by omega-conotoxins known to interact with N-type calcium channels. The discovery of their analgesic properties shed light on the role of N-type calcium channels in pain signal transduction and these channels are now targets for severe pain treatment drugs [15]. Omega-conotoxin MVIIA, also known as ziconotide, is used against chronic pain [16], while many other toxins are in various stages of (pre)clinical trials for the treatment of chronic and neuropathic pain.

Toxin targets and mode of action are key elements to capture in toxin databases. The strategies used to deal with this information differ from one database to the other.

### 5.1. ArachnoServer

In ArachnoServer most manual annotation, including information about the toxin mode of action and targets, is stored in free text in ‘Description’. This description may also include additional topics, such as the presence of a structural domain or sequence similarities to other toxins. Functional information obtained with 100% identical toxins is also annotated. This is the case for kappa-theraphotoxin-Ps1b and kappa-theraphotoxin-Gr2a or mu-agatoxin-Aa1c and mu-agatoxin-Hc1b that have been isolated from different spiders, but have a 100% identical primary structure, and consequently an identical effect on the prey. The fact that identical toxins (most probably orthologs) are named differently raises the issue of nomenclature that will be addressed later in this review.

Information on toxin activity is summarized in ‘Biological Activity’. This section displays three different types of information. First, it associates the activity, if known, with controlled vocabularies, such as ‘Antimicrobial, ‘Cytolytic’, ‘Neurotoxin: Lethal’ or ‘Protease activity’ (e.g., U_17_-ctenitoxin-Pn1a). 15 such terms are available. Second, it deals with molecular targets and provides different measures of toxin’s effectiveness expressed in effective dose (ED(50)) and inhibitory concentration (IC(50)) (e.g., delta-theraphotoxin-Cj1a). Finally, the results of animal testing are reported, including the lethal dose (LD(50)), paralytical dose (PD(50)) and (ED(50)), as well as the name of the tested organism (see for example delta-hexatoxin-Hv1a).

**Figure 5:**
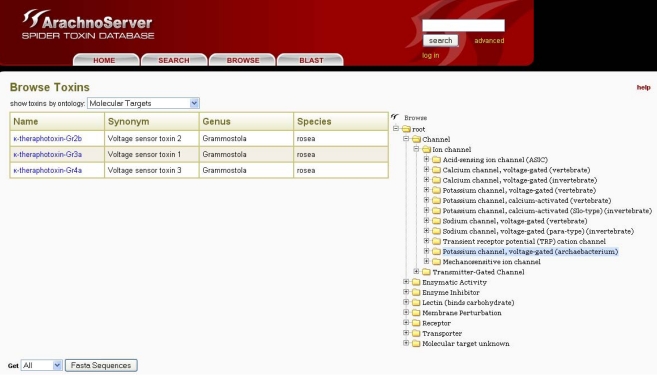
The ArachnoServer browser tool allows toxin retrieval based on their molecular targets.

In ArachnoServer, toxin entries can be accessed by two different approaches: either through the search tab using the toxin name, various characteristics (PTMs, 3D-structure, *etc.*) or biological activities, or with the user-friendly browser tool that permits the use of targets as an entry point (Figure 5). This latter retrieval system is based on a molecular target ontology built on the channel and receptor subtype definitions recommended by the international union of basic and clinical pharmacology (IUPHAR) (see ‘Useful URLs’). It is thus possible to retrieve all spider toxins inhibiting ion channels, for instance, or to restrict the search to one specific channel, such as the voltage-gated calcium channel Cav2.1. 

### 5.2. ConoServer

In ConoServer, description of targets and mode of action is indirectly indicated in the topic ‘Pharmacological family’. In cone snail nomenclature, a Greek letter has been assigned to each pharmacological family, which indicates the group of target ionic channels. For example, ‘omega’ means that the toxin targets are voltage-gated calcium channels. The mapping between Greek letters and targets is provided in the form of lists and of a static tree in the document files of the database (see ‘Useful URLs’). This system does not include any indication concerning precise targets, or experimental evidence. 

### 5.3. UniProtKB

Because UniProtKB is a generalist protein database, it provides immediate access to both toxins and their targets. 

The toxin function is associated with the keyword ‘Toxin’, and sometimes with more specific keywords, such as ‘Cardiotoxin’, ‘Myotoxin’, ‘Neurotoxin’ or ‘Postsynaptic neurotoxin’, which can be used in the search engine. Since this keyword is also used for plant and bacterial toxins, a search for animal toxins should also include ‘metazoa’ in the ‘Taxonomy’ field (e.g., ‘keyword:toxin AND taxonomy:metazoa’). Like ArachnoServer, UniProtKB indicates toxicity, in terms of LD(50), PD(50) and ED(50) in ‘Toxic dose’.

Toxin targets and modes of action are annotated in ‘Function’. Users interested can further search UniProtKB with the target name to find the corresponding entries. UniProtKB/Swiss-Prot uses two nomenclature systems for channel and receptor subtypes, *i.e.*, that of the international union of basic and clinical pharmacology (IUPHAR) (see ‘Useful URLs’) and that of the HUGO Gene Nomenclature Committee (HGNC) [17]. IUPHAR nomenclature is commonly used in literature by neuroscientists, pharmacologists and toxinologists, whereas the HGNC nomenclature is not only the official one for human genes, but also reflects a consensus naming system for all mammalian protein genes.

## 6. Enzymes

While enzymes are present in the majority of venoms [18,19,20,21,22,23,24,25,26,27,28], their number in invertebrates is very limited compared to that in snake venoms. As ArachnoServer and ConoServer deal with invertebrate proteins, data on enzymes are either limited, or nonexistent.

### 6.1. ArachnoServer

At the beginning of November 2009, 47 enzymes were described in ArachnoServer. The Enzyme Commission (EC) number, the reaction in which the enzyme is involved, the family it belongs to, and other related information are annotated in ‘Description’, whereas kinetic parameters, such as Michaelis-Menten constant (K_m_) and maximal velocity (V_max_), are available in ‘Biological Activity’.

ArachnoServer provides links to MEROPS (see ‘Useful URLs’), a database specialized in peptidases.

### 6.2. ConoServer

There are no enzymes in ConoServer. ConoServer focuses on small peptides (10-40 amino acids long) that target membrane bound receptors and ion channels, criteria that most enzymes do not fulfill, although in UniProtKB two such cone snail enzymes are annotated (endoprotease Tex31, Q7YT83 and Conodipine-M, Q9TWL9).

### 6.3. UniProtKB

**Figure 6:**
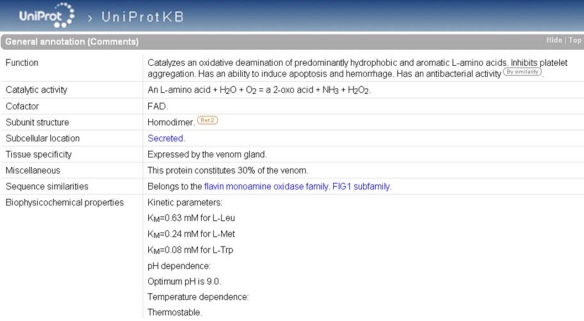
‘General annotation’ section of the L-amino-acid oxidase entry in UniProtKB (accession number P81382). The enzyme-specific annotation is found in the ‘Catalytic activity’, ‘Cofactor’, and ‘Biophysicochemical properties’ subsections.

As is the case in ArachnoServer, enzymes in UniProtKB are characterized by an EC number in the ‘Protein name’. If the catalytic activity is not fully documented, the EC number may be incomplete providing evidence only for the class of the enzyme. A complete EC number in the ‘Protein name’ is always accompanied by a ‘Catalytic activity’ in ‘General annotation (Comments)’ (Figure 6) that describes the enzymatic reaction in a strictly controlled format using controlled vocabulary, following the guidelines of the Nomenclature Committee of the International Union of Biochemistry and Molecular Biology (NC-IUBMB) (see ‘Useful URLs’). Other useful information is available such as:

-    ‘Enzyme regulation’ indicates which factors stimulate or inhibit the reaction (e.g., Q8UVG0),
-    ‘Cofactor’ indicates a cofactor requirement (e.g., Q8I912),
-    ‘Biophysicochemical properties’ provides information on kinetic parameters (K_m_ and V_max_) (e.g., Q5I225), pH (e.g., P0C942) and temperature dependence (e.g., P0C942).

The bibliographic source of the statements, as well as non-experimental qualifiers, may be indicated in ‘General annotation (Comments)’.

Residues directly involved in the catalytic activity are annotated in ‘Sequence annotation (Features)’ under the title ‘Active site’.

For further information, links to specialized enzyme databases such as MEROPS and BRENDA (see ‘Useful URLs’) are available from the ‘Cross-references’ section.

## 7. Nomenclature Issues

Communication or retrieval of information is greatly facilitated by the use of stable common names, approved and used by the whole community. Although this sounds like a truism, toxin nomenclature is still quite far from representing such a simple and unified system.

The majority of toxins are named according to various (and variable) criteria, ranging from the characteristics of the protein, such as PTMs or targets, to technical details of purification. Basically, researchers’ imagination is given free rein to name their favorite toxin. This approach results in often complicated names that provide broad information, but lack ease of use. To illustrate this heterogeneity, we cite alpha-conotoxin-like PuSG1.1 [29], where SG stands for Salivary Gland, or scorpion Tst26, a protein recovered from a column after a 26 minute elution [30].

Currently three nomenclature systems exist, for cone snail, spider and a class of scorpion toxins. 

The cone snail nomenclature proposed by Olivera and others [for a review, see 31] is described in the ConoServer documents (see ‘Useful URLs’). This nomenclature is based on toxin pharmacological activity, disulfide bond connectivity and species of origin. This system is quite complex and is not always followed by the scientific community. In the course of entry creation, ConoServer integrates toxin names found in the literature and stores them in the ‘Name’ or ‘Alternative name(s)’ field.

The recently updated nomenclature for spider toxins is based on toxin activity, taxonomic family and species of origin [2]. Contrary to ConoServer, ArachnoServer renames toxins that do not follow the suggested guidelines, playing thus the role of a nomenclature committee. Additional names found in the literature and abbreviations are stored in a dedicated section called ‘Toxin Synonyms’.

The last nomenclature system concerns the scorpion toxins that specifically target potassium channels. It is based on amino acid sequence motifs and on the location of cysteine residues that are crucial for 3D-structure [32,33].

In UniProtKB, protein names, as well as gene names when available, are stored in ‘Names and origin’. The policy is that toxin names that follow established nomenclature guidelines are stored as ‘Recommended name’. For names not subjected to any nomenclature system the recommended name is left to annotator’s judgment. If possible, the most frequently used name tends to be chosen. Like ConoServer, UniProtKB does not rename toxins. In all cases, synonyms found in the literature or in other resources are kept as ‘Alternative name(s)’.

For the specific case of scorpion potassium channel toxins, UniProtKB proposes a document, called ‘scorpktx.txt’, that lists all toxins known to date, according to the nomenclature system first described in 1999 by Tytgat *et al.* [32] and extended in de la Vega *et al.* [33] and their mapping to the knowledgebase (see ‘Useful URLs’).

MS/MS analysis of venom proteins [1], as well as initiatives to sequence the genomes of venomous animals [34], will accelerate the rate of peptide toxin discovery over the next decade. The need for a standardized toxin nomenclature is becoming urgent in order to facilitate data cataloging, retrieval and especially before databases become too large to allow systematic revision of toxin names. After several attempts and the recent proposal by King *et al.*, to extend the new spider nomenclature to all venomous species [2], the International Society for Toxinology (IST) has established a novel Nomenclature Committee which should help solve this important issue.

## 8. Animal Toxin Database (ATDB)

The Animal Toxin Database (http://protchem.hunnu.edu.cn/toxin/) gathers venom proteins from all venomous animals, as well as their targets [35]. Protein sequences and functional annotation mostly come from UniProtKB, GenPept and three former databases, SCORPION2 [36], the snake neurotoxin database [35] and MOLLUSK [35] whose production has been discontinued. 

**Figure 7:**
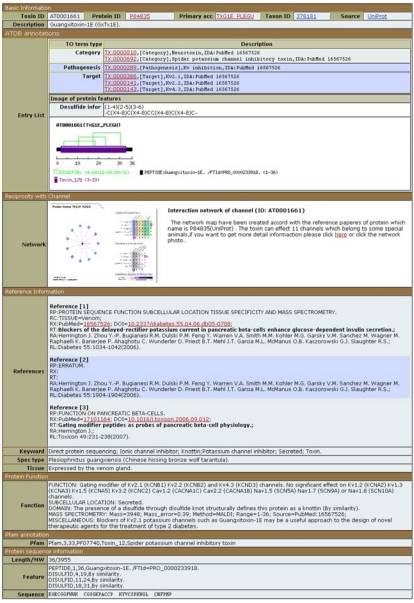
ATDB entry of Guangxitoxin-1E (ATDB accession number AT0001661).

The information unique to ATDB is a toxin ontology and an animal toxin-channel interaction network. The toxin ontology, which includes the modes of action and targets, is displayed at the very top of ATDB entries (Figure 7). The interaction network between the toxin and its targets is shown below and includes not only the interaction itself, but also its effect on the target function (activation, inhibition, *etc.*). Besides these two sections, an ATDB entry provides reformatted information imported from other databases and clearly identifies the origin of the data. Scorpion toxin data are imported mainly from UniProtKB, except for synthetic peptides coming from SCORPION2, a database that is no longer maintained. For snake venom, about 66% of proteins are imported from UniProtKB, and about 2% from the snake neurotoxin database (another database that is no longer maintained). The remaining proteins (about 33%) come from GenPept, the NCBI equivalent of UniProtKB/TrEMBL. For cone snail toxins, about 60% come from UniProtKB and about 40% from the MOLLUSK database. For all other organisms, the information comes from UniProtKB.

ATDB proposes three different systems to find toxins: ‘Search’, ‘Browse’ and ‘Ontology’ tabs. Numerous options are offered to refine searches, to such an extent that a new user may be a little disoriented. 

**Figure 8:**
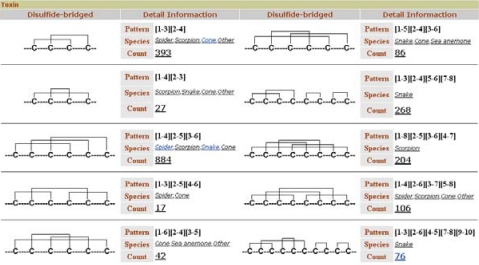
The ATDB browser allows the retrieval of toxins on the basis of their disulfide framework and on the taxonomical group of interest (http://protchem.hunnu.edu.cn/toxin/Browse/Structure.jsp).

Like ConoServer, ATDB permits toxin retrieval based on disulfide connectivity. The browser displays a user-friendly table that depicts the different cystine frameworks in each taxonomical group (Figure 8). This table is accessible from the ‘Browse’ tab, in the topic ‘Toxin’ and the subtopic ‘by Structure’.

Sequence similarity search tools, blastp and blastn, are available, but there are no alignment tools.

## 9. Conclusions

Thanks to their potential pharmacological relevance, animal toxins are in the spotlight of biomedical research. The increase in the amount of data, from sequences to pharmacological characterization, has been quite impressive during the last decade. In this emerging field, only three databases currently acquire experimental data, annotate it and try to provide a global view of the knowledge about these compounds. A fourth database, ATDB, compiles data and annotations from other databases and provides toxin ontology. While the core information (sequences, PTMs, *etc.*) is common to all of these databases, their approaches are quite different. ArachnoServer, and ConoServer, which provide information on spider and cone snail toxins, respectively, are oriented towards the active mature peptides, whereas UniProtKB, a general interest database, displays the longest known product, be it precursor or mature toxin. These different strategies may shed new and complementary light on the field. Users should be aware of these differences and choose the database that suits their needs best. Therefore the coexistence of databases does not imply competition between them, but rather collaboration.

In the coming years, there is no doubt that new toxin databases will be created. Let’s draw up our wish list for the toxin databases’ future. First, researchers must continue to submit nucleotide sequences to the EMBL/GenBank/DDBJ data bank. Such submissions have been regularly done in the past, thanks to publishers who requested an accession number to include in publications. Second, sequences obtained by direct protein sequencing and experimental results should be submitted to UniProtKB, via the SPIN tool (See ‘Useful URLs’). We believe that the policy established for nucleotide sequences should also be applied for protein sequences. In this regard, the support of publishers would be a real asset. This will ensure the best penetration of the data throughout the community. UniProtKB annotates toxins to provide a summary of the current knowledge and play the role of a central hub to direct users to more specialized databases, such as organism-oriented ones, where more detailed information will be provided. This implies the creation of more resources that would reflect the great variety of venomous animals. All resources should work collaboratively to offer a coherent, but complementary view of the field. This process is partially ongoing between the four existing databases and visible at the level of the cross-references they provide. Indeed ConoServer and ArachnoServer provide cross-references to UniProtKB, UniProtKB provides cross-references to ArachnoServer and is currently implementing cross-references to ConoServer. As the only general interest database, UniProtKB could play the role of a central hub for information on toxins, providing basic annotation and redirecting users to the appropriate resources for more specialized data. Such collaboration may help in finding the right balance between specialized annotation and integration of the toxin field within the broad picture of life sciences.

**Table 1:** Summary table.

	ArachnoServer	ConoServer	UniProtKB/Swiss-Prot	ATDB (metadatabase)
Topic	Spider toxins	Conotoxins	General interest, includes animal toxins and their targets	Animal toxins and their targets
**Sequences**	Mature peptides and precursors	Mature peptides and precursors	Longest available protein sequences (precursors or mature sequences) (nucleotide sequences available via cross-references to EMBL/GenBank/DDBJ)	Protein sequences (as in source database), and some nucleotide sequences
	Nucleotide sequences	Nucleotide sequences		
Tools	BLAST	Sequence search, Fingerprint Search	BLAST, ProtParam, Compute pI/MW, ProtScale	BLAST
		Clustal alignment, Digest Peptide, Analyse prosequence	PeptideMass, PeptideCutter	
Cross-references	EMBL, UniProtKB	GenBank, GenPept, UniProtKB	EMBL/GenBank/DDBJ	GenBank, GenPept, UniProtKB, SCORPION2 (obsolete)
			ArachnoServer	Snake neurotoxin database (obsolete)
			ConoServer (work-in-process)	MOLLUSK (obsolete)
Sequence download	In FASTA format	In FASTA format	In FASTA format	In FASTA format
**PTMs **	Experimental PTMs (direct and indirect evidences indicated)	Experimental PTMs (disulfide bonds only indicated in cysteine framework)	Experimental PTMs (direct and indirect evidences indicated)	As in source database.
Search	Via the search tool or the browser	Only disulfide bonds (via the cysteine framework)	Via the search tool	Only disulfide bonds
**3D-structure**	Shown if available	Shown if available	Available via cross-references to PDBsum and SMR (3D modeling)	Shown if available
Cross-references	PDB	PDB	PDB	PDB
		BMRB	PDBsum	
			SMR	
**Functions and Targets**	In ‘Description’ (free text) and ‘Biological Activity’ (controlled vocabulary)	In ‘Classification’, a Greek letter indicates the target	In ‘General annotation (free text) and ‘Ontologies’ (controlled vocabulary)	In ‘Description’ using ‘Toxin ontology’ terms (controlled vocabulary).
				As in source database (free text)
Toxin effectiveness	In ‘Biological activity’.	Not indicated	In ‘General annotation’	As in source database
Animal testing (LD(50), ED(50), PD(50))	In ‘Biological activity’.	Not indicated	In ‘General annotation’	As in source database
Channel and receptor nomenclature	IUPHAR	Not indicated	IUPHAR	IUPHAR
			HGNC (only for human proteins and propagated to mammalian orthologs)	HGNC(only for mammalian proteins and propagated to mammalian orthologs)
**Enzymes**	In ‘Description’ (free text) and ‘Biological Activity’.	none	In ‘General annotation’	As in source database
			(EC number in ‘Protein names’)	
Active site and Metal binding	Not indicated		In ‘Sequence annotation (Features)’	As in source database
Cross-references	MEROPS		MEROPS	
			BRENDA	
**Nomenclature**	Creation of new names and names from the literature	Names from the literature	Names from the literature	As in source database

## Useful URLs

ArachnoServer (http://www.arachnoserver.org/)

ConoServer (http://www.conoserver.org/):

Document on conopeptide nomenclature:http://www.conoserver.org/?page=about_conotoxins&bpage=cononames
Document on conopeptide targets:http://www.conoserver.org/index.php?page=about_conotoxins&bpage=conotargets
Document on conopeptide PTMs:http://www.conoserver.org/?page=about_conotoxins&bpage=ptm

UniProtKB (http://www.uniprot.org/):

Direct submissions to UniProtKB: http://www.ebi.ac.uk/swissprot/Submissions/spin/
Description of the Tox-Prot annotation program: http://www.expasy.org/sprot/tox-prot/
Keyword list: http://www.uniprot.org/docs/keywlist
Document on scorpion toxin nomenclature: http://www.uniprot.org/docs/scorpktx
Document on cross-references: http://www.uniprot.org/docs/dbxref
Protein nomenclature publication list: http://www.uniprot.org/docs/nomlist
  PROTPARAM: http://www.expasy.org/tools/protparam-doc.html
  PI Tool: http://www.expasy.org/tools/pi_tool-doc.html
  Protscale: http://www.expasy.org/tools/protscale-doc.html
  Peptide mass: http://www.expasy.org/tools/peptide-mass-doc.html
  Peptidecutter: http://www.expasy.org/tools/peptidecutter/peptidecutter_instructions.html

ATDB (http://protchem.hunnu.edu.cn/toxin/)

BMRB (http://www.bmrb.wisc.edu/)

BRENDA (http://www.brenda-enzymes.org)

IUBMB (http://www.chem.qmul.ac.uk/iubmb/enzyme/)

IUPHAR (http://www.iuphar.org/)

MEROPS (http://merops.sanger.ac.uk/)

PDB (http://www.pdb.org/)

PDBsum (http://www.ebi.ac.uk/pdbsum/)
